# Comparison of hepatitis B viral loads and viral antigen levels in child-bearing age women with and without pregnancy

**DOI:** 10.1186/s12884-018-1932-9

**Published:** 2018-07-06

**Authors:** Chenyu Xu, Jingli Liu, Lanhua Liu, Yongchun Bi, Biyun Xu, Jie Chen, Biao Xu, Tingmei Chen, Yali Hu, Yi-Hua Zhou

**Affiliations:** 1 Department of Obstetrics and Gynecology, Zhenjiang Fourth People’s Hospital, Zhenjiang, 212001 Jiangsu China; 2 Departments of Laboratory Medicine and Infectious Diseases, Nanjing Drum Tower Hospital and Jiangsu Key Laboratory for Molecular Medicine, Nanjing University Medical School, 321 Zhongshan Road, Nanjing, 210008 Jiangsu China; 3 Department of Obstetrics and Gynecology, Taixing People’s Hospital, Taixing, 225400 Jiangsu China; 4 Department of Biostatistics, Nanjing Drum Tower Hospital, Nanjing University Medical School, Nanjing, 210008 Jiangsu China; 5 Department of Obstetrics and Gynecology, Nanjing Drum Tower Hospital, Nanjing University Medical School, Nanjing, 210008 Jiangsu China; 6 Department of Infectious Diseases, Nanjing Drum Tower Hospital, Nanjing Medical University, Nanjing, 210008 Jiangsu China

**Keywords:** Pregnancy, HBV replication, Expression of HBsAg and HBeAg

## Abstract

**Background:**

Pregnancy is a unique physiological condition with the cellular immune functions compromised at some extents to allow the mature of growing fetus. Whether pregnancy may influence the replication of hepatitis B virus (HBV) is less studied. The present study aimed to investigate the influence of pregnancy on the replication of HBV and expression of viral antigens by comparing the levels of HBV DNA and viral antigens in pregnant and non-pregnant women.

**Methods:**

A total of 727 HBsAg-positive serum samples, collected from 214 pregnant women and 513 non-pregnant women of childbearing age, were included. Based on the pregnancy status, subjects were divided into four groups: nulliparous (*n* = 158), pregnant (*n* = 214), 7–12 months postpartum (*n* = 170), and 2–5 years postpartum (*n* = 185). The levels of hepatitis B surface antigen (HBsAg) and hepatitis B e antigen (HBeAg) were quantitatively measured with microparticle enzyme immunoassay. HBV DNA levels were detected by fluorescent real-time PCR.

**Results:**

The median ages of four groups were 25.0, 25.3, 26.2 and 29.3 years, respectively (*p* < 0.01). HBeAg-positive proportions were 34.2, 33.6, 35.3 and 29.2%, respectively (*p* = 0.624). HBV DNA levels in HBeAg-positive women were higher than those in HBeAg-negative women (7.88 vs 2.62 log IU/ml, *p* < 0.001). HBV DNA levels in the four groups with positive HBeAg were 7.8, 7.7, 8.0 and 8.0 log IU/ml, respectively (*p* = 0.057), while HBsAg titers were 4.4, 4.5, 4.6 and 4.8 log IU/ml (*p* = 0.086) and HBeAg titers were 3.1, 3.0, 3.1 and 3.0 log S/CO (*p* = 0.198). In the four groups with negative HBeAg, HBV DNA levels were 2.3, 2.6, 2.5 and 2.8 log IU/ml, respectively (*p* = 0.085), while HBsAg titers were 3.1, 3.3, 3.3 and 3.0 log IU/ml (*p* = 0.06).

**Conclusions:**

Serum levels of HBV DNA and viral antigens showed no significant changes in nulliparous, pregnant, and postpartum women, regardless of the HBeAg status. The results indicate that pregnancy has little influence on the replication of HBV and the expression of viral antigens.

## Background

Chronic hepatitis B virus (HBV) infection is a serious health problem because of the severe sequelae such as liver cirrhosis and cancer. It is estimated that there are approximately 250 million chronic carriers worldwide [1]. Transmission of HBV from carrier mothers to their children is an important cause of chronic infection in hepatitis B endemic areas. Before the availability of hepatitis B immunoglobulin (HBIG) and hepatitis B vaccine, 10–30% and 70–90% of the children born to HBV carrier mothers with negative hepatitis B e antigen (HBeAg) and to HBeAg-positive carrier mothers had been chronically infected with HBV respectively. Measures for preventing mother-to-child transmission of HBV, combined use of HBIG and hepatitis B vaccine [2, 3], have been implemented in most countries. The combined immunoprophylaxis measures are highly effective; nearly zero and < 10% transmission occurred in children born to HBeAg-negative and HBeAg-positive carrier mothers respectively [4–7]. More recently, antiviral therapy in late pregnancy in pregnant women with high viral load is increasing [8, 9], although the safety of antiviral drugs remains further observations [10–12]. On the other hand, the influence of pregnancy on the replication of HBV in pregnant women infected with HBV is less studied. A survey in Massachusetts General Hospital, Boston, USA, showed a high frequency of inadequate care of mothers with chronic hepatitis B post-pregnancy [13].

Pregnancy is a unique physiological condition. A successful pregnancy requires the tolerance of maternal immune system to the growing fetus as a fetus is actually a semi-allogeneic graft, in which half of the genomes are derived from the father. Pregnancy represents a period of time with particular changes of immunological functions, compromised at some extents to allow the mature of a fetus [14]. Usually, the tolerance of immune response occurs in the cellular immune arm. Pregnant women are susceptible to reactivation and infection of latent viruses, such as herpes zoster virus and cytomegalovirus [15, 16], or elevated replication level of infected virus, such as hepatitis C virus [17]. It is generally considered that the replication of HBV is increased during pregnancy due to the immunosuppression [18], but relevant studies are limited and the conclusions are inconsistent [19, 20]. The present study compared the levels of HBV DNA and viral antigens in pregnant women with those in non-pregnant women to clarify whether the replication of HBV is increased during pregnancy.

## Methods

### Study subjects

Totally 727 women who were positive for hepatitis B surface antigen (HBsAg) and had normal alanine aminotransferase levels were enrolled from October 2009 to March 2014. The median age was 26.0 (22.0–32.4) years old. All women were negative for antibodies against hepatitis C virus and human immunodeficiency virus. Those who had history of antiviral therapy or were taking antiviral drug were excluded. Additionally, the liver function tests of these women were basically normal, in which both alanine transaminase and aspartate transaminase were < 50 IU/L (1.25 upper normal limitation) and total bilirubin levels were < 21 μmol/L. Based on the pregnancy and reproductive history, the women were divided into four groups: 158 women who never had history of pregnancy in group A (nullipara), 214 women who were in the third trimester (from gestation 28 weeks to just before delivery) in group B (pregnancy), 170 women at 7–12 months postpartum in group C (puerperant), and 185 women at 2–5 years postpartum in group D (puerperant). The peripheral blood samples were collected and serum samples were separated by centrifugation and frozen at − 30 °C. All samples were sent to Nanjing Drum Tower Hospital in ice box for the laboratory testing, including quantification of HBV DNA and viral antigen and genotyping.

This study, including the form of informed consent, was approved by the ethics committee of each of the three participating hospital, including Nanjing Drum Tower Hospital (approval no. 2012019), Zhenjiang Fourth People’s Hospital (approval no. 2011–425), and Taixing People’s Hospital (approval no. 2012–266). The study was performed in accordance with the ethical standards in the Declaration of Helsinki. Written informed consent was obtained from each woman.

### Detection of hepatitis B serological markers and HBV DNA

Serological markers of hepatitis B, including hepatitis B surface antigen (HBsAg), HBeAg, antibody against HBeAg (anti-HBe) and antibody against hepatitis B core antigen (anti-HBc), were qualitatively detected with commercial enzyme-linked immunosorbent assay reagents (Shanghai Kehua Bio-Engineering Co. Ltd., Shanghai, China. Products numbers: HBsAg, YBS00192010; HBeAg, 20,163,400,144; anti-HBe, YZB/2138–2012; anti-HBc 20,163,400,143). The measurements were performed according to the manufacturer’s instructions, with using 50 μl serum for detecting HBsAg, HBeAg, and anti-HBe respectively, and 50 μl 30-fold diluted serum (1.7 μl serum and 48.3 μl normal solution) for detecting anti-HBc. The levels of HBsAg and HBeAg were further quantitatively measured with microparticle enzyme immunoassay reagents (ARCHITECT, Abbott, North Chicago, USA); when the HBsAg levels were higher than the upper detection limit (250 IU/ml), the sera were diluted to 1:20 to 1:1000 to obtain a reading within the range of the calibration curve. All the measurements of quantification of HBsAg and HBeAg were completed within one day with same batch reagents by an investigator (YB) who was unaware of the serum identity.

Serum HBV DNA levels were quantitatively detected with real-time fluorescence quantitative PCR reagents (Shenyou Biotechnology, Shanghai, China; Products numbers, YZB/0080–2009) on a thermal cycler (Applied Biosystems 7500 Real-time PCR System, Singapore), with the detection limit 100 IU/ml. To minimize the variation in measuring HBV DNA levels, all samples were measured with same batch reagents by the same investigator (JL) who was blinded to sample identity and the test was performed strictly according to the manufacturer’s instructions.

### HBV genotyping

The serum DNA was extracted from 200 μl serum with the proteinase K digestion and phenol/chloroform extraction method and dissolved in 20 μl Tris-EDTA buffer as previously reported [21, 22]. The S gene was amplified with nested polymerase chain reaction (PCR) with the primers C1 (nt300–318, 5’-YTGGCCAAAATTCGCAGTC-3′) and C2 (nt1017–998, 5’-AAACCCCARRAGACCCACAA-3′) for the first round amplification, and C3 (nt325–342, 5’-CTCCARTCACTCACCAAC-3′ and C4 (nt992–973, 5’-TGACAKACYTTCCAATCAAT-3′) for the second amplification. The resultant PCR products were purified by PCR purification kit (Tiangen Biotech, Beijing, China), followed by thermocycling reaction using BigDye Terminator v3.1 sequencing reagents (ABI, USA), and then directly sequenced on an ABI 3130 Gene Analyzer. The S sequences were aligned with reference sequences that included genotypes A to H, which were obtained from the NCBI website (URL: http://www.ncbi.nlm.nih.gov/projects/genotyping/view.cgi?db=2). HBV genotypes were determined by phylogenetic analysis based on the S gene sequences as previously described [22].

### Statistical analysis

The statistical software SPSS 11.0 (SPPS Inc., Chicago, IL, USA) was used for the analysis. Inter-group comparison was carried out after the quantitative levels of HBV DNA, HBsAg and HBeAg were converted to logarithmic values. Measurement data was descried with median and range, inter-group differences were analyzed with Kruskal-Wallis test, and then Nemenyi post-hoc test was used to analyze the difference between any two groups. Categorical data were described with rate and inter-group differences were analyzed with χ^2^ test or the Fisher exact test. A *p* value < 0.05 with two-tailed test was considered statistically significant.

## Results

### Demographic and virological characteristics in women

The median age of women in each group and proportion of women with positive HBeAg were presented in Table 1. The age difference between nullipara and pregnancy groups had no statistical significance, while the age in puerperant 7–12 months postpartum and puerperant 2–5 years postpartum groups was slightly higher than that in two other groups (*p* < 0.05). The HBeAg positive proportion in the four groups was comparable, with the overall proportion of 33.0% (Table 1). The median serum HBV DNA level in women with positive HBeAg was significantly higher than that in those with negative HBeAg (7.88 vs 2.62 log IU/ml, *p* < 0.001). HBV genotypes were determined in 689 women and were not determined in 38 other women due to the undetectability of HBV DNA (Table 1). The main genotypes were B and C. Overall, compared with the nulliparous and parturient women, the pregnant women had no increased serum levels of HBV DNA and HBsAg (Table 1).

**Table 1:** Demographic and virological characteristics in 727 HBsAg-positive pregnant women and women with different reproductive histories

	Total (*n* = 727)	Nullipara (*n* = 158)	Pregnancy (*n* = 214)	Puerperant, 7–12 months postpartum (*n* = 170)	Puerperant, 2–5 years postpartum (*n* = 185)	*p*
Age (year)	26.0 (22.0–32.4)	25.0 (22.0–30.0)	25.3 (22.7–30.2)	26.2 (23.6–31.5)^a^	29.3 (24.0–32.4)^a, b^	< 0.05
Positive HBeAg (%)	240 (33.0)	54 (34.2)	72 (33.6%)	60 (35.3)	54 (29.2)	0.624
HBV DNA, log IU/ml (min-max)	3.1 (1.4–9.2)	3.0 (1.4–9.0)	3.1 (1.6–8.9)	3.2 (1.5–9.2)	3.1 (1.4–9.1)	0.543
HBsAg, log IU/ml (min-max)	3.7 (0.9–5.9)	3.4 (0.9–5.0)	3.7 (0.9–5.9)	3.8 (1.0–5.4)	3.5 (1.3–5.4)	0.072
Genotype^c^						
B (%)	303 (44.2)	59 (44.4)	70 (33.5)^d^	77 (46.7)	97 (54.2)	0.001
C (%)	383 (55.8)	74 (55.6)	139 (66.5)^d^	88 (53.3)	82 (45.8)	

The units of numbers in the parentheses are indicated in the first column

*HBeAg* hepatitis B e antigen, *HBsAg* hepatitis B surface antigen, *HBV* hepatitis B virus

^a^*p* < 0.05 compared with the age of nulliparas and pregnant women

^b^*p* < 0.05 compared with puerperant women 7–12 months postpartum

^c^HBV genotype was not determined in 38 women because of failure in the amplification of HBV DNA, including 24 nulliparas, 4 pregnant women, 5 puerperants 7–12 months postpartum and 5 puerperants 2–5 years postpartum. Additionally, there were two women infected with genotype D and one woman infected with genotype G. Since the numbers for genotypes D and G were too low to be meaningfully compared, we did not include these three women in the analysis

^d^*p* = 0.043, 0.010, and < 0.001 compared with the frequencies of genotypes B and C in nullipara group, puerperant 7–12 months postpartum and puerperant 2–5 years postpartum respectively. The differences between any other groups had no statistical significance (all *p* > 0.05)

### Comparison of levels of HBV DNA and viral antigens in carrier women with positive HBeAg

The presence of HBeAg indicates the active replication of HBV. To rule out the potential influence of different proportions of HBeAg positivity in the four groups of women on the viral replication, we separately compared the levels of HBV DNA and viral antigens in HBeAg-positive and -negative women. As shown in Table 2, the virological characteristics and the frequency of genotypes were similar among the four groups. The genotypes were exclusively B and C, which is in agreement with the findings in China [23]. There was no statistically significant difference in serum HBV DNA level between pregnant women and non-pregnant women or women after childbirth (*p* = 0.057). Similarly, serum HBsAg and HBeAg concentrations were each comparable among different groups respectively.

**Table 2:** Comparison of virological characteristics in 240 HBeAg-positive pregnant women and women with different reproductive history

	Nullipara (*n* = 54)	Pregnancy (*n* = 72)	Puerperant, 7–12 months postpartum (*n* = 60)	Puerperant, 2–5 years postpartum (*n* = 54)	*p*
Age (year)	25.0 (22.0–29.0)	23.8 (22.7–30.6)	25.6 (23.6–31.0)	28.9 (24.0–32.3)^a^	< 0.05
HBV DNA, log IU/ml (min-max)	7.8 (1.6–9.0)	7.7 (1.7–8.9)	8.0 (1.6–9.2)	8.0 (2.9–9.1)	0.057
HBsAg, log IU/ml (min-max)	4.4 (2.8–5.0)	4.5 (2.7–5.9)	4.6 (2.2–5.4)	4.8 (1.9–5.4)	0.086
HBeAg, log S/CO (min-max)	3.1 (0.7–3.3)	3.0 (1.4–3.3)	3.1 (0.7–3.2)	3.0 (1.2–3.5)	0.198
Genotype^b^					
B (%)	18 (34.0)	24 (33.3)	25 (41.7)	16 (29.6)	0.579
C (%)	35 (66.0)	48 (66.7)	35 (58.3)	38 (70.4)	

*HBeAg* hepatitis B e antigen, *HBsAg* hepatitis B surface antigen, *HBV* hepatitis B virus

^a^*p* < 0.05 compared with the age of three other groups

^b^HBV genotype was not determined in one woman in nullipara group due to the undetectability of HBV DNA

To exclude the potential influence of extremely high or low HBV DNA levels in some women on the overall viral levels, we analyzed the frequency of high (≥6 log IU/ml), moderate (3–< 6 log IU/ml) and low (< 3 log IU/ml) serum HBV DNA levels in the four groups of HBeAg positive women (Table 3). The frequencies of high, moderate and low viral levels had no significant difference in the four groups.

**Table 3:** The frequency of high, moderate, and low level of HBV DNA in 240 carrier women with positive HBeAg

HBV DNA (log IU/ml)	Nullipara (*n* = 54)	Pregnancy (*n* = 72)	Puerperant, 7–12 months postpartum (*n* = 60)	Puerperant, 2–5 years postpartum (*n* = 54)	*p*
< 3 (%)	3 (5.5)	2 (2.8)	3 (5.0)	1 (1.9)	0.684
3–< 6 (%)	7 (13.0)	4 (5.5)	5 (8.3)	3 (5.5)	0.449
≥6 (%)	44 (81.5)	66 (91.7)	52 (86.7)	50 (92.6)	0.226

HBeAg, hepatitis B e antigen; HBV, hepatitis B virus. The units of numbers in the parentheses are indicated in the first column

### Comparison of HBV DNA and HBsAg levels in HBV carrier women with negative HBeAg

Negativity of HBeAg, regardless of the positivity of anti-HBe, indicates the low replication of HBV. We compared the levels of HBV DNA and HBsAg in the four groups of carrier women with negative HBeAg. Table 4 shows that the levels of HBV DNA in the pregnant women were comparable with those in the nulliparous women, puerperants 7–12 months and 2–5 years postpartum, respectively. The frequency of high (≥6 log IU/ml), moderate (3–< 6 log IU/ml) and low (< 3 log IU/ml) levels of HBV DNA in these four groups were comparable (Table 5). Similarly, the levels of HBsAg in the four groups of women had no significant difference (Table 4).

**Table 4:** Virological characteristics in HBeAg-negative carrier women in 487 pregnant women and women with different pregnant histories

	Nullipara (*n* = 104)	Pregnancy (*n* = 142)	7–12 months postpartum (*n* = 110)	2–5 years postpartum (*n* = 131)	*p*
Age (year)	25.0(22.0–30.0)	25.0 (22.8–30.3)	27.0 (23.7–31.2)^a^	29.5 (24.3–32.3)^a, b^	< 0.05
HBV DNA, log IU/ml (min-max)	2.3 (1.4–6.6)	2.6 (1.6–6.7)	2.5 (1.5–7.0)	2.8 (1.4–6.0)	0.085
HBsAg, log IU/ml (min-max)	3.1 (0.9–4.8)	3.3 (0.9–4.2)	3.3 (1.0–4.3)	3.0 (1.3–4.2)	0.06
Genotype^c^					
B (%)	41 (51.3)	46 (33.6)^d^	52 (49.5)^e^	81 (64.8)	< 0.001
C (%)	39 (48.7)	91 (66.4)^d^	53 (50.5)^e^	44 (35.2)	

*HBeAg* hepatitis B e antigen, *HBsAg* hepatitis B surface antigen, *HBV* hepatitis B virus

^a^*p* < 0.05 compared with the age of nulliparas and pregnant women

^b^*p* < 0.05 compared with the age of puerperant women, 7–12 months postpartum

^c^The HBV genotype was not determined in 37 women because HBV DNA level was too low to be detected, including 23 nulliparas, 4 pregnant women, 5 puerperants 7–12 months postpartum and 5 puerperants 2–5 years postpartum. Additionally, there were one woman infected with genotype D and another woman infected with genotype G. Since the numbers for genotypes D and G were too low to be meaningfully compared, we did not include these three women in the table

^d^*p* = 0.010, 0.012, and < 0.001 compared with the frequencies of genotypes B and C in nullipara group, puerperant 7–12 months postpartum and puerperant 2–5 years postpartum respectively

^e^*p* = 0.019 compared with the frequencies of genotypes B and C in puerperant 2–5 years postpartum. The differences between any other groups had no statistical significance (all *p* > 0.05)

**Table 5:** The frequency of high, moderate and low level of HBV DNA in 487 carrier women with negative HBeAg

HBV DNA, log IU/ml	Nullipara (*n* = 104)	Pregnancy (*n* = 142)	7–12 months postpartum (*n* = 110)	2–5 years postpartum (*n* = 131)	*p*
< 3 (%)	71 (68.3)	99 (69.7)	72 (65.5)	87 (66.4)	0.890
3–< 6 (%)	31 (29.8)	41 (28.9)	37 (33.6)	43 (32.8)	0.821
≥6 (%)	2 (1.9)	2 (1.4)	1 (0.9)	1 (0.8)	0.900

*HBeAg* hepatitis B e antigen, *HBV* hepatitis B virus

## Discussion

The data in the present study show that the levels of HBV DNA and viral antigens in the pregnant women were comparable with those in nulliparas, puerperants 7–12 months and 2–5 years postpartum, respectively, indicating that the replication of HBV and expression of viral antigens during pregnancy are not increased in spite of the presence of immune tolerance during pregnancy.

In individuals infected with HBV, the proportion of positive HBeAg is decreased with age. In East Asia, the positive HBeAg proportion in HBV carrier women aged 20–39 years is 27.3–36.1% [24]. In the present study, the age distribution in the participant is relatively homogenous (22.0–32.4 years old). Only the women 2–5 years postpartum had the relatively higher age, and the proportion of positive HBeAg was somewhat decreased (Table 1), which is in agreement with the findings reported in the literature [24]. The positivity of HBeAg indicates active viral replication. When the Abbott reagents were used, the median HBV DNA level in HBeAg-positive women was 8.1 log IU/ml, much higher than 2.7 log IU/ml in HBeAg-negative women [25]. When Roche reagents were used, the average HBV DNA level in HBeAg-positive women was 7.4 log copies/ml, much higher than 2.7 log copies/ml in HBeAg-negative ones [26]. In the present study, serum HBV DNA level was measured by the reagents made in China, and the results showed that pregnant women with positive and negative HBeAg was 7.88 and 2.62 log IU/ml, respectively, with a difference of 5.26 log IU/ml. Additionally, the frequencies of high (≥6 log IU/ml), moderate (3–< 6 log IU/ml), and low (< 3 log IU/ml) viral loads detected in the present study (Tables 3 and 5) were also consistent with the reported results assayed with Abbott and Roche reagents [25, 26]. These results suggest that the reagents for quantification of HBV DNA made in China are also reliable. Thus, the data in the present study reflected the real HBV DNA levels in these women.

It is reported that different HBV genotypes may vary in some extent in the viral replication [23]. B and C are the main HBV genotypes in China, and it is generally considered that viral load is higher in patients infected with genotype C than that in those infected with genotype B [23]. Among HBeAg positive women in this study, the frequencies of genotypes B and C were similar among pregnant and non-pregnant groups respectively (Table 2). In HBeAg negative women, the frequency of genotype C was the highest in pregnant women (Table 4). However, the levels of HBV DNA and HBsAg in the pregnant women remained to be comparable to the levels in three other non-pregnant women (Tables 2 and 4). Therefore, the slight difference in the frequencies of genotypes in the pregnant and non-pregnant women should have minimal influence on the HBV DNA levels in these women.

Generally, placental trophoblast cells can secrete various cytokines and hormones during pregnancy to maintain pregnant women in an immunosuppressive status by regulating major histocompatibility complex (MHC) or Th1/Th2 balance. For example, MHC-I and II molecules are not expressed on trophoblast cells, which are not recognized by maternal immune system. Meanwhile, the expression of human leukocyte antigen G can inhibit the activity of natural killer cells and T cells [27]. In addition, indoleamine 2,3-dioxygenase secreted by trophoblast cells can inhibit the proliferation of T cells by degrading tryptophan [28]. The elevation of estrogen and progesterone during pregnancy may also be involved in the immunosuppression in pregnant women [29]. The immunosuppressive status during pregnancy makes pregnant women be predisposed to reactivation of latent viral infections, such as cytomegalovirus, polyomavirus, and human papillomavirus [12–17, 30, 31]. However, since the levels of HBV DNA in the circulation may reflect the replication of HBV in hepatocytes, the findings that the levels of HBV DNA, HBsAg, and HBeAg in pregnant women are each comparable with those in the non-pregnant women in the present study indicate that the immunosuppression during pregnancy does not result in the enhanced replication of HBV.

The direct evidence to clarify whether pregnancy has influence on the replication of HBV is to longitudinally observe the changes of viral loads and antigen levels before and during pregnancy and postpartum in a same cohort of pregnant women infected with HBV. However, the low feasibility of recruiting HBV-infected women just prior to conception limited us to perform the longitudinal study. This is the main limitation of the present study. Additionally, the serum samples were collected from the women who delivered their babies and from female patients who visited our hospitals, which might be biased. However, we included a large number of HBV-infected women with similar proportion of HBeAg positive women in each group. The findings that pregnant women did not show higher viral loads and viral antigen levels than the non-pregnant women may at least provide indirect evidence that pregnancy has no influence on the replication of HBV.

Pregnant women infected with HBV are more predisposed to have abnormal liver function tests than pregnant women without HBV infection, but the liver function tests in the women included in the present study were basically normal, which is the another limitation in this study. However, the observations that pregnancy does not activate the replication of HBV suggest that liver injury in pregnant women infected with HBV is less likely related to the reactivation of viral replication, which merits further study.

## Conclusion

Based on the large numbers of participating women infected with HBV in an endemic area, serum levels of HBV DNA and viral antigens in pregnant women showed no significant changes compared with those in nulliparous and postpartum women. The results indicate that pregnancy has minimal influence on the replication of HBV and the expression of viral antigens.

## Abbreviations

anti-HBe: Antibody against HBeAg
HBeAg: Hepatitis B e antigen
HBIG: Hepatitis B immunoglobulin
HBsAg: Hepatitis B surface antigen
HBV: Hepatitis B virus
MHC: Histocompatibility complex
PCR: Polymerase chain reaction
